# Emergence and Expansion of a Carbapenem-Resistant Pseudomonas aeruginosa Clone Are Associated with Plasmid-Borne *bla*_KPC-2_ and Virulence-Related Genes

**DOI:** 10.1128/mSystems.00154-21

**Published:** 2021-05-18

**Authors:** Yanyan Hu, Congcong Liu, Qi Wang, Yu Zeng, Qiaoling Sun, Linbin Shu, Jiayue Lu, Jiacang Cai, Shaolin Wang, Rong Zhang, Zuowei Wu

**Affiliations:** a Department of Clinical Laboratory, Second Affiliated Hospital, Zhejiang University School of Medicine, Hangzhou, China; b College of Veterinary Medicine, China Agricultural University, Beijing, China; c Department of Veterinary Microbiology and Preventive Medicine, Iowa State University, Ames, Iowa, USA; UC Davis Genome Center

**Keywords:** *Pseudomonas aeruginosa*, ST463, carbapenem resistance, clonal expansion, nosocomial infections

## Abstract

Pseudomonas aeruginosa is a major opportunistic pathogen and one of the leading bacterial species causing health care-associated infections. Carbapenems are the most effective antimicrobial agents for the treatment of severe infections caused by P. aeruginosa. However, our recent surveillance demonstrated that the prevalence of carbapenem-resistant P. aeruginosa (CRPA) reached 38.67% in Zhejiang, China. By analyzing CRPA isolates collected from patients from 2006 to 2018, we found that 33% of CRPA isolates carried the gene *bla*_KPC-2_, which conferred high-level resistance to carbapenems and other β-lactams. In particular, a CRPA clone, ST463 (sequence type 463), emerged and has become the predominant CRPA clone among the population. Genome sequencing demonstrated that ST463 expansion was associated with plasmid-borne *bla*_KPC-2_. The mobile element flanking *bla*_KPC-2_, the type IV secretion system, and the successful expansion of clone ST463 might have further favored *bla*_KPC-2_ spread in P. aeruginosa. Molecular clock analysis dated the emergence of clone ST463 to around 2007. Genome-wide association analysis showed that 567 genes were associated with clone ST463, including several known virulence genes related to the biosynthesis of lipooligosaccharide (LOS) O-antigens and exotoxin. These findings indicate that ST463 is expanding with plasmid-borne *bla*_KPC-2_ and virulence-related genes in nosocomial infections, and close surveillance should be undertaken in the future.

**IMPORTANCE** Health care-associated infections, also known as nosocomial infections, are the most frequent adverse events in health care delivery worldwide, causing high rates of morbidity and mortality and high health care costs. Pseudomonas aeruginosa is one of the leading bacterial species causing health care-associated infections. Carbapenems are the most effective antimicrobial agents for the treatment of its severe infections. However, the prevalence of carbapenem-resistant P. aeruginosa (CRPA) has been increasing rapidly in recent years, and our surveillance demonstrated that the prevalence of CRPA reached 38.67% in Zhejiang, China. Genome sequencing of CRPA isolates over a decade showed that a CRPA clone (ST463) emerged recently. The clone is highly resistant to β-lactams, including carbapenems, and fluoroquinolones. Genome-wide association analysis showed that the clone expanded with virulence-related genes and the plasmid-borne carbapenem-resistant gene *bla*_KPC-2_. These findings are of significant public health importance, as the information will facilitate the control and minimization of CRPA nosocomial infections.

## INTRODUCTION

Pseudomonas aeruginosa is a major opportunistic pathogen and one of the leading bacterial species causing health care-associated infections (1). P. aeruginosa is commonly associated with pneumonia, bloodstream infections, urinary tract infections, and surgical site infections and causes substantial morbidity and mortality (2, 3). According to the U.S. Centers for Disease Control and Prevention (CDC), P. aeruginosa annually causes an estimated 51,000 health care-associated infections with roughly 400 deaths in the United States (4). Because of its intrinsic resistance and the high capability to acquire resistance genes, P. aeruginosa is resistant to a range of antimicrobial agents, which often makes infections difficult to treat (5). Carbapenems are the most effective agents for the treatment of severe P. aeruginosa infections (6). However, the prevalence of carbapenem-resistant P. aeruginosa (CRPA) has increased rapidly in recent years (7). In 2016, the World Health Organization (WHO) ranked CRPA as one of the top critical pathogens in health care settings (8). In China, CHINET surveillance showed that the rates of resistance of P. aeruginosa to imipenem and meropenem in 2019 were 27.5% and 23.5%, respectively (http://www.chinets.com/). CRPA has posed a significant threat to public health worldwide.

The mechanisms of resistance to carbapenems among P. aeruginosa strains are multifactorial (9). Generally, P. aeruginosa can become resistant to carbapenems by the acquisition of plasmids or integron-mediated carbapenemases, such as the metallo-β-lactamases (MBLs), Klebsiella pneumoniae carbapenemases (KPCs), and GES enzymes (10–12), which are important due to their high efficiency in hydrolyzing carbapenems. In addition, several other mechanisms can confer low levels of carbapenem resistance. For example, the repression or inactivation of the carbapenem porin OprD and the hyperexpression of the chromosomal cephalosporinase AmpC are associated with reduced susceptibility to carbapenems (13–15). The overexpression of efflux pump systems such as MexAB-OprM also contributes to meropenem resistance (16). These mechanisms alone or together confer P. aeruginosa resistance to carbapenems.

P. aeruginosa is diverse genetically and has a nonclonal epidemic population structure (17, 18). However, several multilocus sequence types (STs) have spread worldwide and are frequently associated with epidemics where multidrug resistance confounds treatment. These so-called “international” or “high-risk” clones include ST111, ST175, ST235, and ST395 (19). The global success of these clones is expected to be determined by a complex interplay between pathogenicity, epidemicity, and antibiotic resistance. Recent studies suggested that ST235 most likely emerged as a global clone due to a unique combination of virulence genes (*exoU* and a *dprA*-related gene) and the ability to readily acquire antibiotic resistance genes that limit antibiotic treatment options (18), and the copper resistance of ST395 may have accounted for its spreading capability in hospital outbreaks (20). In our hospital in Zhejiang, China, we recently detected an emergent clone (ST463) of P. aeruginosa that is highly resistant to carbapenems and other β-lactam antibiotics (21). In this study, we analyzed CRPA isolates from 2006 to 2018 and found that ST463 has become predominant in the CRPA population and is highly resistant to β-lactams, including carbapenems, and fluoroquinolones. Genome sequencing and association analyses indicate that the emergence and expansion of ST463 are associated with plasmid-borne *bla*_KPC-2_ and virulence-related genes.

## RESULTS

### Prevalence of KPC-producing P. aeruginosa (*bla*_KPC-2_ CRPA) in a Chinese hospital.

**FIG 1:**
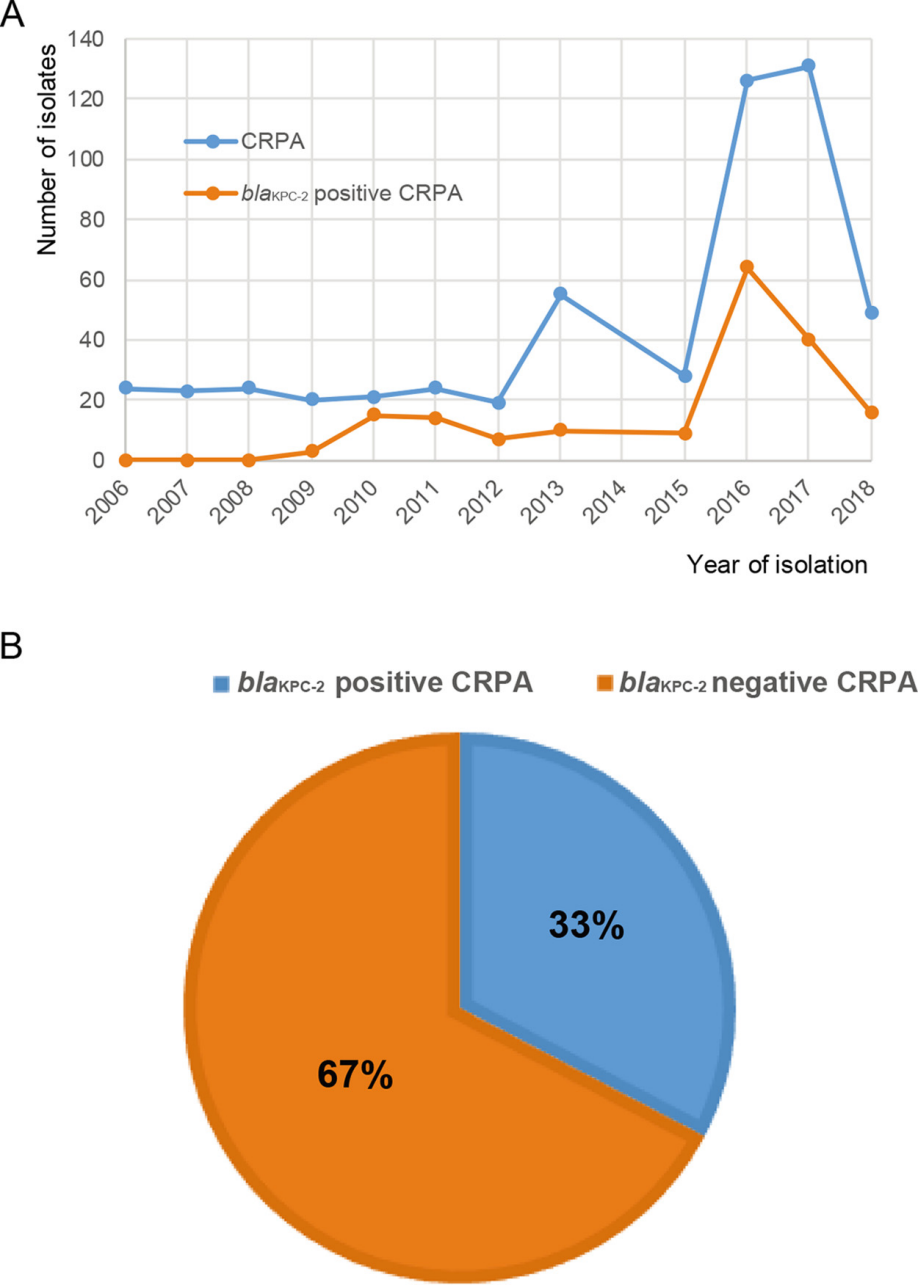
Prevalence of *bla*_KPC-2_-mediated carbapenem-resistant P. aeruginosa (CRPA) from 2006 to 2018 in a hospital in Zhejiang, China. (A) Yearly distribution of CRPA and *bla*_KPC-2_ CRPA isolates investigated in this study. (B) Average prevalence of *bla*_KPC-2_-mediated CRPA.

Carbapenems are the most effective antimicrobial agents against severe nosocomial infections involving P. aeruginosa that produces the cephalosporinase AmpC or extended-spectrum β-lactamases (ESBLs). P. aeruginosa has been increasingly becoming resistant against carbapenems (7). Our recent hospital surveillance showed that CRPA reached a prevalence of 38.9%, similar to the levels (38.67%) demonstrated in the province of Zhejiang, China (22). In this study, the gene *bla*_KPC-2_ was detected by PCR from 544 CRPA isolates that were collected from patients in our hospital from 2006 to 2018. The gene *bla*_KPC-2_ was initially detected in CRPA isolates collected in 2009 and has become prevalent since then (Fig. 1A). In total, 33% (*n* = 180) of the tested CRPA isolates carried the carbapenem resistance gene *bla*_KPC-2_ (Fig. 1B). Clinically, the *bla*_KPC-2_-positive CRPA isolates were commonly associated with the respiratory tract, surgical wounds, and the urinary tract of intensive care unit (ICU) patients, posing severe threats to the patients (see Table S1 in the supplemental material).

10.1128/mSystems.00154-21.4 TABLE S1 Clinical information on 143 carbapenem-resistant Pseudomonas aeruginosa isolates. Download Table S1, XLSX file, 0.02 MB. Copyright © 2021 Hu et al. 2021 Hu et al. https://creativecommons.org/licenses/by/4.0/ This content is distributed under the terms of the Creative Commons Attribution 4.0 International license.

### ST463 predominates in the *bla*_KPC-2_ CRPA population.

**FIG 2:**
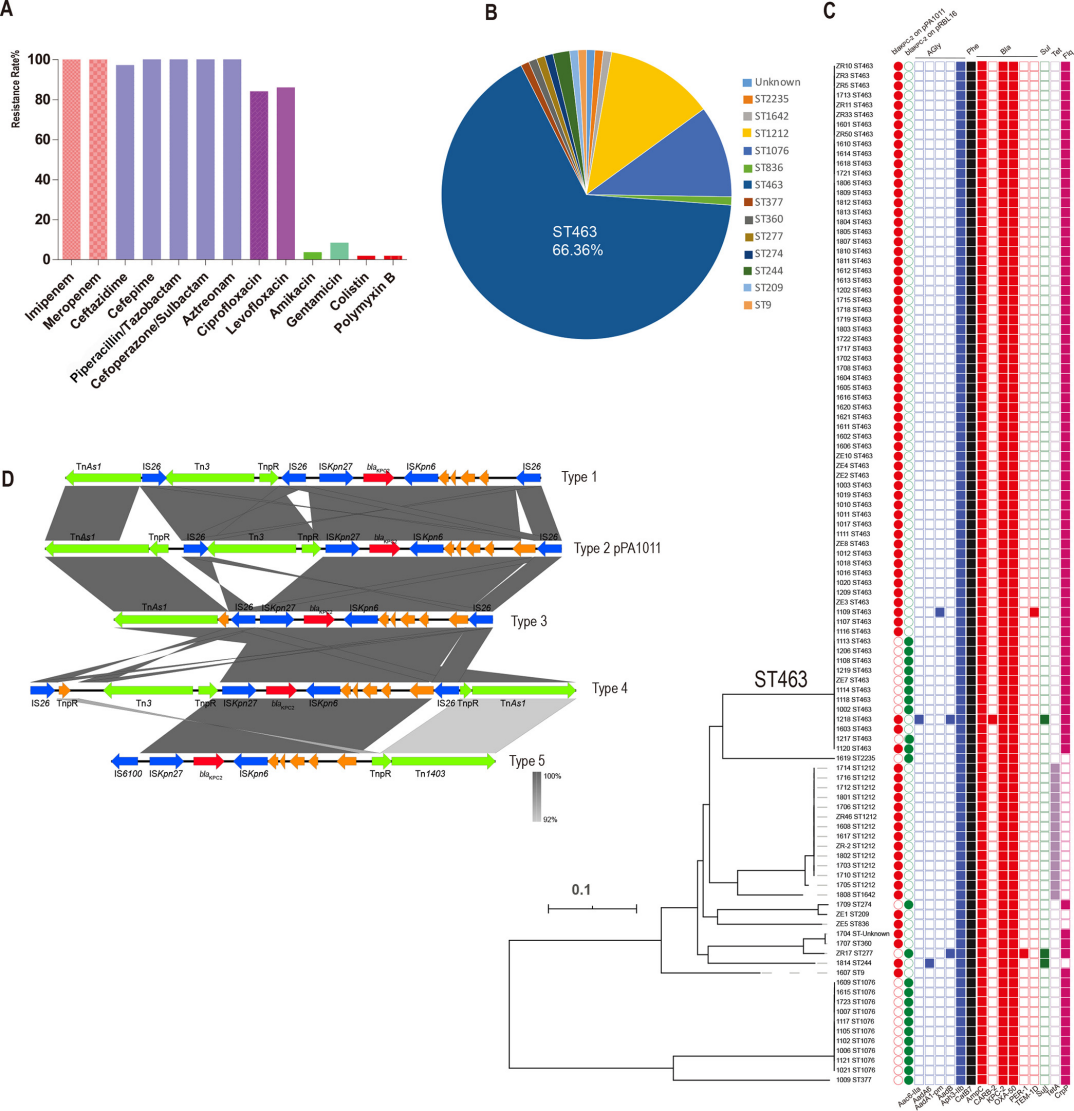
Antimicrobial resistance and genomic analysis of 105 *bla*_KPC-2_-carrying carbapenem-resistant P. aeruginosa (CRPA) isolates. (A) Rates of resistance of *bla*_KPC-2_ CRPA to 13 routinely used antimicrobial agents for the treatment of P. aeruginosa in clinical settings. (B) Sequence types in *bla*_KPC-2_ CRPA isolates. ST463 is the predominant ST (66.36%). (C) *bla*_KPC-2_ location and resistance genes. A maximum likelihood phylogenetic tree was constructed using the core-genome SNPs of the 105 isolates and midpoint rooted, showing that ST463 is highly clonal (bar, number of nucleotide substitutions per site). The location of *bla*_KPC-2_ on plasmids pPA1011 and/or pRBL16 is denoted by filled circles (red, pPA1011; green, pRBL16). The presence and absence of antibiotic resistance genes are denoted by filled and empty squares, respectively. Resistance categories are differentiated by colors. (D) The diverse genetic context of *bla*_KPC-2_. Tnp, ISs, *bla*_KPC-2_, and hypothetical proteins are indicated by green, blue, red, and orange, respectively. Five different types were identified, but the IS*Kpn27*-*bla*_KPC-2_-IS*Kpn6* unit was conserved in all the isolates.

To characterize the phenotypes and genotypes of *bla*_KPC-2_-positive CRPA isolates, 105 out of 180 *bla*_KPC-2_ CRPA isolates were chosen randomly from our collection spanning from 2009 to 2018. Antimicrobial susceptibility testing showed that the *bla*_KPC-2_ CRPA isolates were highly resistant to β-lactams, including carbapenems, and fluoroquinolones. However, low-level resistance to aminoglycosides and polymyxins was demonstrated among the *bla*_KPC-2_ CRPA isolates tested (Fig. 2A and Table 1). Multilocus sequence typing (MLST) analysis revealed 13 STs and an unknown ST among the 105 *bla*_KPC-2_ CRPA isolates tested (Fig. 2B). ST463 was the dominant sequence type, accounting for 66.36% (Fig. 2B and C). Genome sequence analysis identified 15 acquired resistance genes in total in the 105 isolates tested (Fig. 2C). Among them, *aph(3′)-IIb*, *catB7*, *ampC*, and *bla*_OXA-50_ are chromosomally located genes in P. aeruginosa and were detected in all isolates tested (15, 23–25). The other 11 acquired genes included 4 aminoglycoside resistance genes, 4 β-lactam resistance genes, 1 sulfate resistance gene, 1 tetracycline resistance gene, and 1 fluoroquinolone resistance gene. However, the prevalence of these 11 acquired genes was highly variable. *crpP* was present in 82.9% of isolates (87 out of 105), and *tetA* was present in 13.3% of isolates (14 out of 105). The other 9 acquired genes were present in only a few isolates (*n* = 1 to 3). Among the 4 acquired β-lactam resistance genes, *bla*_KPC-2_ was the most prevalent (100%). In contrast, the genes *bla*_CARB-2_, *bla*_PER-1_, and *bla*_TEM-1D_ were detected in only three separate isolates, resulting in three isolates encoding *bla*_KPC-2_ with the ESBL gene *bla*_CARB-2_, *bla*_PER-1_, or *bla*_TEM-1D_ (Fig. 2C).

**TABLE 1:** Antimicrobial susceptibilities of 105 *bla*_KPC-2_-positive CRPA and 38 *bla*_KPC-2_-negative CRPA isolates

Antibiotic(s)	*bla*_KPC-2_-positive CRPA (*n* = 105)			*bla*_KPC-2_-negative CRPA (*n* = 38)			Total resistance rate (%) (*n* = 143)
	Resistance rate (%)	MIC_50_ (μg/ml)	MIC_90_ (μg/ml)	Resistance rate (%)	MIC_50_ (μg/ml)	MIC_90_ (μg/ml)	
Imipenem	100	256	>256	100	16	128	100
Meropenem	100	>256	>256	100	16	64	100
Ceftazidime	99.0	64	128	48.7	8	128	84.2
Cefepime	100	>256	>256	76.9	16	256	93.8
Piperacillin/tazobactam	100	256	>256	71.8	64	>256	92.5
Cefoperazone/sulbactam	100	>256	>256	74.4	64	>256	93.2
Aztreonam	100	>256	>256	74.4	16	256	93.2
Ciprofloxacin	84.1	4	32	48.7	1	32	66.4
Levofloxacin	86	16	64	76.9	4	32	83.6
Amikacin	3.7	1	2	28.2	2	>64	10.3
Gentamicin	8.4	2	4	38.5	2	>64	16.4
Colistin	1.9	1	1	7.7	1	2	3.4
Polymyxin B	1.9	1	2	2.6	1	2	2.1

### The spread of *bla*_KPC-2_ is mediated by plasmids.

*bla*_KPC-2_ was first reported in Klebsiella pneumoniae (26). It is usually located in plasmids with mobile elements (i.e., insertion sequences [ISs] and transposons [tnp]), making *bla*_KPC-2_ highly transferable in the hospital environment (27). In the 105 isolates, two reference plasmids were detected by PlasmidSeeker (28) from the Illumina reads, including pPA1011 (GenBank accession number MH734334) and pRBL16 (GenBank accession number CP015879.1). The presence of the plasmid in each isolate was further confirmed by analyzing the presence of the gene from each reference plasmid (Fig. S1). pPA1011 is a 62,793-bp plasmid from a carbapenem-resistant P. aeruginosa strain, which was first reported in our hospital and carried the carbapenem resistance gene *bla*_KPC-2_ (29). pRBL16 is a 370,338-bp plasmid and was originally reported in Pseudomonas citronellolis SJTE-3 isolated from active sludge of a wastewater treatment plant in China but did not carry the resistance gene *bla*_KPC-2_ (30). To determine the location of the gene *bla*_KPC-2_ in the 105 isolates, *bla*_KPC-2_-containing contigs were extracted from Illumina assemblies and aligned to pPA1011 and pRBL16. The comparison of the flanking sequences of *bla*_KPC-2_ showed that *bla*_KPC-2_ was carried by pPA1011 and/or pRBL16. Out of 104 isolates, 81 carried *bla*_KPC-2_ on pPA1011, and 23 carried *bla*_KPC-2_ on pRBL16 (Table S2). One isolate, 1120 of ST463, was found to contain two copies of the gene *bla*_KPC-2_ via Nanopore sequencing. One was carried by pPA1011, and the other one was carried by pRBL16 (Fig. 2C). The mobile genetic elements and antibiotic resistance genes in proximity to *bla*_KPC-2_ were analyzed, after annotation of the *bla*_KPC-2_-containing contigs. In the 105 isolates, *bla*_KPC-2_ coexisted with the chromosomally located resistance genes (*aph3-IIb*, *catB7*, *ampC*, and *bla*_OXA-50_) and/or other resistance genes. Interestingly, no antibiotic resistance genes coexisted with *bla*_KPC-2_ in the same contig, indicating that those resistance genes may be on the chromosome or not in the immediate proximity of *bla*_KPC-2_ in the plasmid. In pPA1011, *bla*_KPC-2_ was flanked immediately by IS*Kpn27* (upstream) and IS*Kpn6* (downstream). In addition, *bla*_KPC-2_ was flanked by several mobile elements, including Tn*As1*, IS*26*, Tn*3*, and TnpR further upstream and IS*26* further downstream (Fig. S2); however, the mobile genetic elements were not conserved among the 105 *bla*_KPC-2_ CRPA isolates (Fig. S1). To further characterize the genetic context of *bla*_KPC-2_, eight isolates were chosen for long-read sequencing (Nanopore) according to the absence patterns of these mobile elements from Illumina sequencing (Fig. S1). By analyzing the flanking genes in each *bla*_KPC-2_-containing contig from Illumina and Nanopore assemblies, the patterns of mobile elements in the *bla*_KPC-2_ genetic context were classified into five types (Fig. 2D), indicating the diverse genetic context of *bla*_KPC-2_. The immediately flanking mobile elements of *bla*_KPC-2_ were present in all 105 isolates tested, forming a conserved IS*Kpn27*-*bla*_KPC-2_-IS*Kpn6* genetic context (Fig. 2D), for both pPA1011 and pRBL16. *bla*_KPC-2_ in pRBL16 has the same flanking genetic environment in the isolates tested, characteristic of the *bla*_KPC-2_ insertion between the genes A9C11_RS32220 (hypothetical protein) and A9C11_RS32225 (hypothetical protein). pPA1011 encoded a type IV secretion system (T4SS), which is related to conjugation, making it potentially conjugatable and transferable.

10.1128/mSystems.00154-21.1 FIG S1 Detected genes of reference plasmids pPA1011 (GenBank accession number MH734334) and pRBL16 (GenBank accession number CP015879.1) in the 143 carbapenem-resistant Pseudomonas aeruginosa isolates. The presence and absence of genes are denoted by filled and empty circles, respectively. Download FIG S1, PDF file, 0.7 MB. Copyright © 2021 Hu et al. 2021 Hu et al. https://creativecommons.org/licenses/by/4.0/ This content is distributed under the terms of the Creative Commons Attribution 4.0 International license.

10.1128/mSystems.00154-21.2 FIG S2 Circular representation of plasmid pPA1011. The mobile elements, *bla*_KPC-2_, and the type IV secretion system on the plasmid are shown in the outermost circle. Download FIG S2, PDF file, 0.4 MB. Copyright © 2021 Hu et al. 2021 Hu et al. https://creativecommons.org/licenses/by/4.0/ This content is distributed under the terms of the Creative Commons Attribution 4.0 International license.

10.1128/mSystems.00154-21.5 TABLE S2 Genome assembly stats and characterization of resistance genes and virulence genes in 143 carbapenem-resistant Pseudomonas aeruginosa isolates. Download Table S2, XLSX file, 0.3 MB. Copyright © 2021 Hu et al. 2021 Hu et al. https://creativecommons.org/licenses/by/4.0/ This content is distributed under the terms of the Creative Commons Attribution 4.0 International license.

### *bla*_KPC-2_-negative CRPA isolates are diverse genetically.

**FIG 3:**
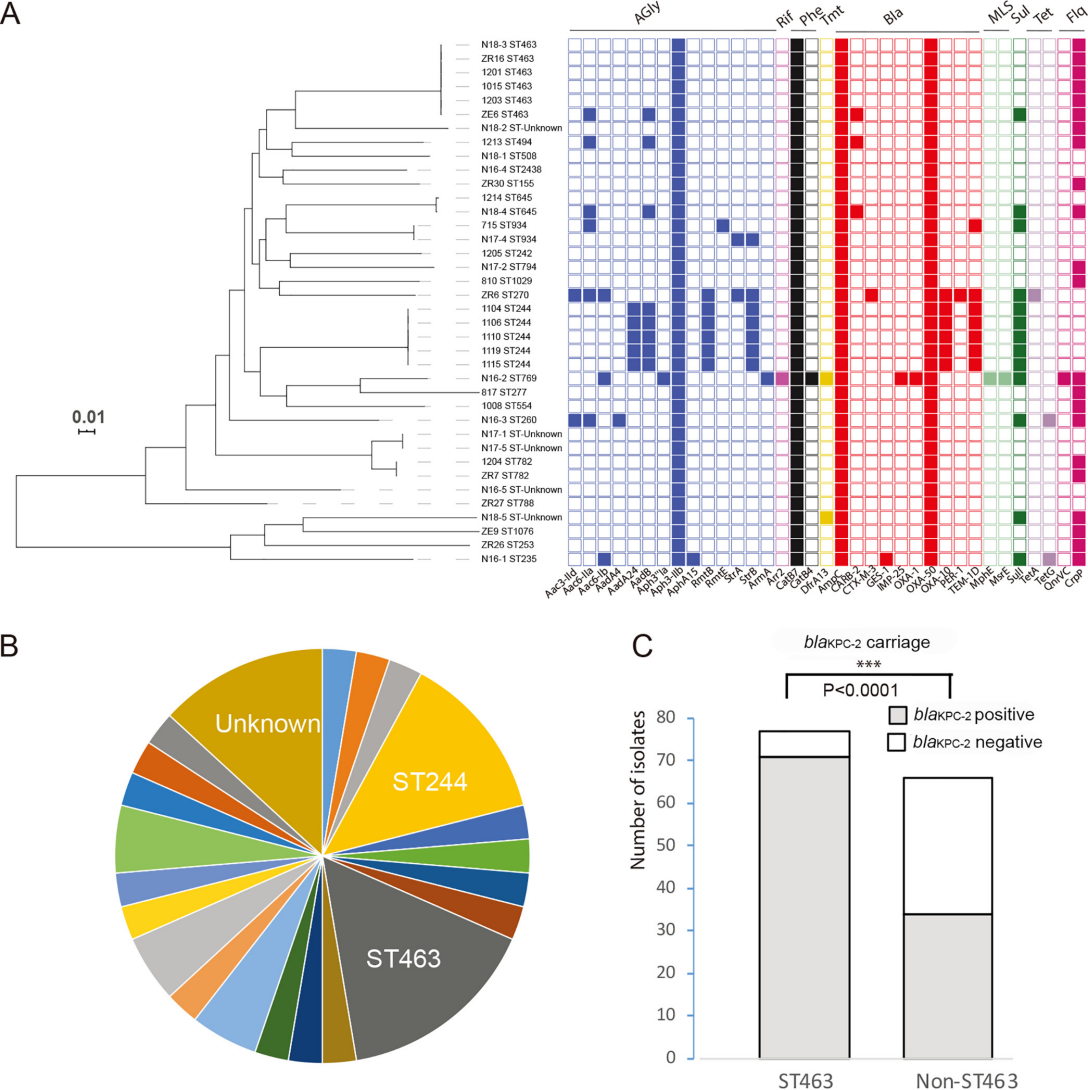
Genomic analysis of 38 *bla*_KPC-2_-negative carbapenem-resistant P. aeruginosa (CRPA) isolates. (A) Resistance genes. A maximum likelihood phylogenetic tree was constructed using the core-genome SNPs and midpoint rooted (bar, number of nucleotide substitutions per site). The presence and absence of antibiotic resistance genes are denoted by filled and empty squares, respectively. Resistance categories are differentiated by colors. (B) Sequence types in the *bla*_KPC-2_-negative CRPA isolates. No predominant ST was identified. (C) Comparison of *bla*_KPC-2_ carriage between ST463 and non-ST463 isolates (Fisher’s exact test). *bla*_KPC-2_ and ST463 are highly associated (71 out of 77; 92.2%).

To characterize the phenotypes and genotypes of *bla*_KPC-2_ PCR-negative CRPA isolates, a total of 38 isolates were chosen randomly from our collection spanning from 2009 to 2018. Antimicrobial susceptibility testing showed that the *bla*_KPC-2_-negative CRPA isolates tested were highly resistant to carbapenems and intermediately resistant to other β-lactams, fluoroquinolones, and aminoglycosides but had low-level resistance to polymyxins (Table 1). MLST analysis of these isolates revealed 21 STs in 33 isolates, and 5 isolates did not belong to known STs. There was no dominant ST (ST463 accounted for only 15.7% [Fig. 3B]). Sequence analysis identified 35 resistance genes in total among the 38 isolates (Fig. 3A). Among them, the chromosomally located resistance genes *aph3-IIb*, *catB7*, *ampC*, and *bla*_OXA-50_ were present in all 38 isolates. The other 31 acquired genes included 13 aminoglycosides resistance genes, 1 rifampin resistance gene, 1 phenicol resistance gene, 1 trimethoprim resistance gene, 8 β-lactam resistance genes, 2 macrolide-lincosamide-streptogramin resistance genes, 1 sulfonamide resistance gene, 2 tetracycline resistance genes, and 2 fluoroquinolone resistance genes. However, the prevalences of these 31 genes were highly variable. *crpP* was present in 57.9% of isolates (22 out of 38). The others were each present in only a few isolates (*n* = 1 to 8). Similar to the *bla*_KPC-2_-positive CRPA isolates, the β-lactam resistance genes, except *bla*_OXA-50_, were each detected in one to seven isolates (Fig. 3A). Antibiotic susceptibility testing demonstrated that the MIC_50_s of carbapenems and other β-lactams for *bla*_KPC-2_-positive CRPA isolates were 4- to 16-fold higher than those for *bla*_KPC-2_-negative CRPA isolates, although both *bla*_KPC-2_-positive and -negative CRPA isolates were resistant (Table 1). KPC β-lactamases, encoded by *bla*_KPC-2_, hydrolyze β-lactams of all classes and are highly efficient in hydrolyzing carbapenem antibiotics (31). The high prevalence of *bla*_KPC-2_-positive CRPA isolates has significantly enhanced the levels of resistance to carbapenems and other β-lactams.

### The recent emergence and clonal expansion of ST463 are associated with plasmid-borne *bla*_KPC-2_ and virulence-related genes.

**FIG 4:**
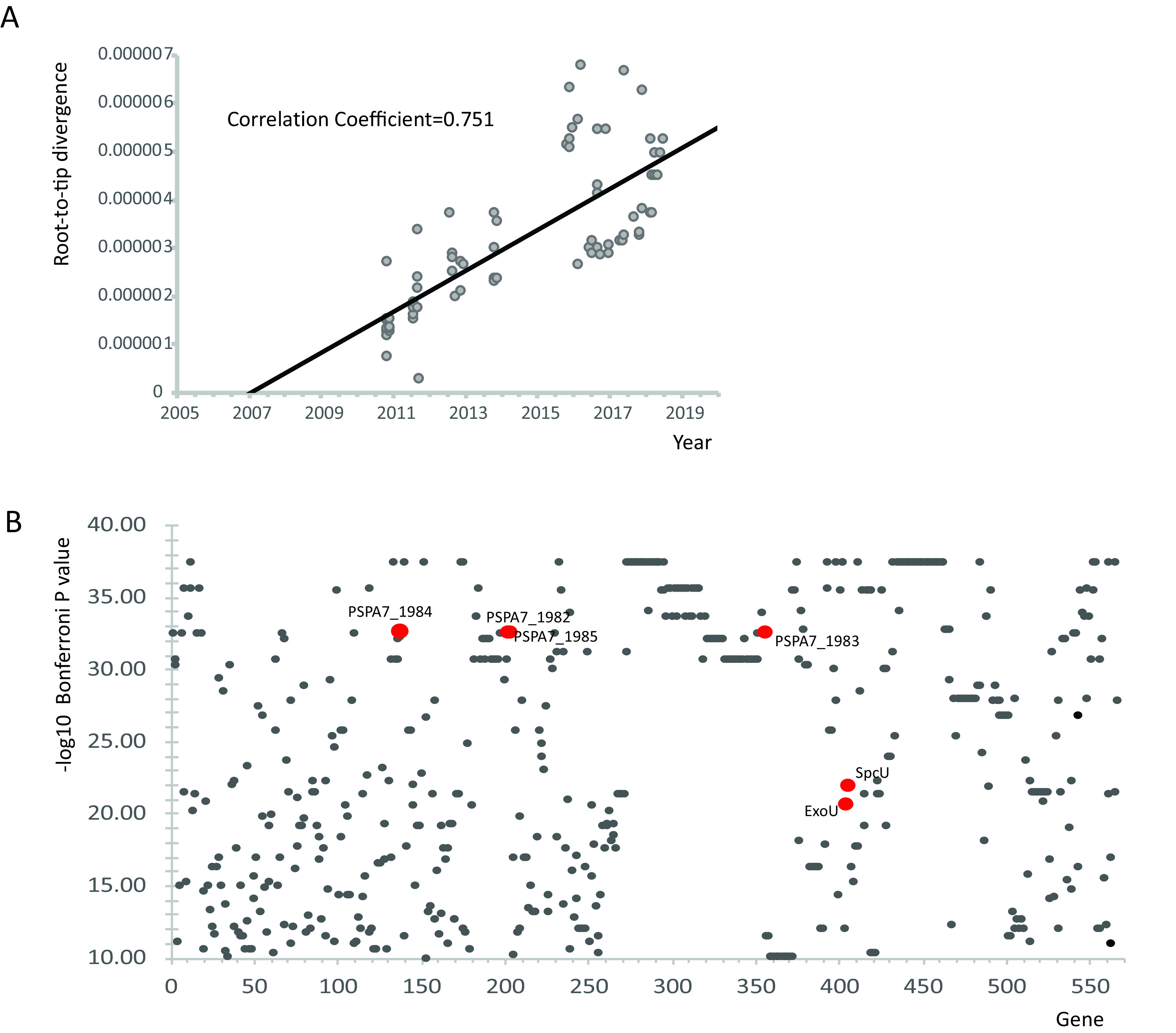
Population genetics analysis of P. aeruginosa ST463 isolates. (A) Regression analysis between isolation dates (*x* axis) and root-to-tip distance (*y* axis). The analysis was conducted based on core genomes of 77 ST463 isolates. The point where the line intersects with the *x* axis gives the inferred date when the most recent common ancestor of ST463 emerged. (B) Genome-wide association analysis of ST463 isolates. The Manhattan plot shows the 567 genes with a significant positive association with ST463. The *x* axis shows the gene identifiers (see Table S3 in the supplemental material), and the *y* axis represents the negative logarithm of the Bonferroni-adjusted *P* value (i.e., the significance) of the association. The known virulence genes are indicated in red.

CRPA isolates were demonstrated to be genetically diverse (30 known STs and 6 unknown STs). However, ST463 accounted for the majority of the CRPA population collection (53.85%), and similarly, this occurred in the *bla*_KPC-2_-positive CRPA isolates tested (66.36%). ST463 was initially detected in our collection in 2009. Core-genome phylogenetic analysis showed that ST463 isolates clustered closely and originated monophyletically (Fig. 2C). Based on the 5,904,108-site alignment of the genomes of 77 ST463 isolates (71 KPC-2 positive and 6 KPC-2 negative), temporal Bayesian analyses (BEAST) inferred a common ancestor in 2007 with a mutation rate of ∼5.446 × 10^−7^ substitutions per site per year. Regression analysis revealed a strong linear relationship between the number of single nucleotide polymorphisms (SNPs) and isolation dates (*R* = 0.751) (Fig. 4A). The above-described analysis indicated a recent emergence and clonal expansion of ST463. Further subtyping of ST463 isolates by whole-genome SNP and whole-genome MLST (wgMLST) analyses did not identify any dominant subtypes, which indicated that ST463 has expanded clonally (Fig. S3).

10.1128/mSystems.00154-21.3 FIG S3 Subtyping of 77 ST463 isolates by whole-genome SNP (A), cgMLST (B), and wgMLST (C) analyses. Each dot indicates a subtype, and the subtypes including multiple isolates are shown by colors. No predominant subtypes are identified among the ST463 isolates. Download FIG S3, PDF file, 0.4 MB. Copyright © 2021 Hu et al. 2021 Hu et al. https://creativecommons.org/licenses/by/4.0/ This content is distributed under the terms of the Creative Commons Attribution 4.0 International license.

10.1128/mSystems.00154-21.6 TABLE S3 Genome-wide association study (GWAS) identified genes associated with Pseudomonas aeruginosa ST463. Download Table S3, XLSX file, 0.1 MB. Copyright © 2021 Hu et al. 2021 Hu et al. https://creativecommons.org/licenses/by/4.0/ This content is distributed under the terms of the Creative Commons Attribution 4.0 International license.

In this study, CRPA isolates were collected from patients, and 90.6% of the patients (131 out of 143) had been treated with β-lactam and/or carbapenem antibiotics prior to bacterial isolation (Table S1). Interestingly, almost all the analyzed ST463 isolates (71 out of 77; 92.2%) carried the gene *bla*_KPC-2_, which was significantly higher than the other genotypes (Fig. 3C). This suggested that the expansion of ST463 is associated with *bla*_KPC-2_ carriage. The high-level resistance to carbapenems and other β-lactams mediated by *bla*_KPC-2_ may have favored the adaptation and survival of ST463 after high levels of usage of these antibiotics during clinical treatment of nosocomial infections, as carbapenems and other β-lactams are clinically important antibiotics and are commonly used in ICU (32).

To detect other genes associated with ST463, a genome-wide association analysis was performed. In total, 567 genes were associated with clone ST463 (Fisher’s exact test *P* value of <0.05 and Bonferroni-adjusted *P* value of <1E−10) (Fig. 4B). The associated genes were involved in broad functions such as cell wall/membrane/envelope biogenesis, transcriptional regulation, inorganic ion transport and metabolism, defense mechanisms, amino acid transport and metabolism, lipid transport and metabolism, and cell motility. These bacterial genes are important to combat stress within hosts, such as immune system surveillance, interspecies competition, and nutrient scarcity (33). Interestingly, among the 567 ST463-associated genes, 6 genes (*PSPA7_1982*, *PSPA7_1983*, *PSPA7_1984*, *PSPA7_1985*, *exoU*, and *spcU*) are known virulence genes (34). *PSPA7_1982*, *PSPA7_1983*, *PSPA7_1984*, and *PSPA7_1985* are located in the serotype island of P. aeruginosa and are involved in the biosynthesis of lipooligosaccharide (LOS) O-antigens (35). ExoU and its chaperone SpcU are components of the P. aeruginosa type III secretion system. The secretion of ExoU has been associated with more severe infections in both humans and animal models (36, 37). However, 81% of these 567 ST463-associated genes remain functionally unknown (Table S3). Overall, ST463 expanded recently with plasmid-borne *bla*_KPC-2_ and important virulence-related genes.

## DISCUSSION

Nosocomial infections caused by P. aeruginosa have become a health care concern globally, mainly due to the high level of resistance to antibiotics. Eight categories of antimicrobial agents are frequently used to treat P. aeruginosa infections, including aminoglycosides, carbapenems, cephalosporins, fluoroquinolones, penicillins with β-lactamase inhibitors, monobactams, phosphonic acids, and polymyxins. Unfortunately, P. aeruginosa can easily develop resistance through chromosomal mutations or by the horizontal acquisition of resistant determinants against the antimicrobials commonly used in the treatment of P. aeruginosa infections (38). As we found in this study, P. aeruginosa isolates were highly resistant to multiple drugs, including carbapenems (imipenem and meropenem), other β-lactams (ceftazidime, cefepime, piperacillin/tazobactam, cefoperazone/sulbactam, and aztreonam), and fluoroquinolones (ciprofloxacin and levofloxacin), but relatively susceptible to aminoglycosides (amikacin and gentamicin) and polymyxins (colistin and polymyxin B) (Table 1). This study reiterated the limited options of antibiotics for the treatment of nosocomial infections caused by P. aeruginosa.

Carbapenems are usually used as last-resort drugs for the treatment of infections caused by multiresistant P. aeruginosa due to their broad spectrum of antibacterial activity and high efficacy (7). Chromosomal mutations (such as those leading to the loss or inactivation of the OprD porin and/or the overexpression of efflux pumps) are the common mechanisms of carbapenem resistance in P. aeruginosa, but the acquisition of carbapenemase-encoding genes is extremely relevant because they are highly efficient in hydrolyzing carbapenems and other β-lactams (39). In this study, genome analysis identified 11 β-lactamases (AmpC, CARB-2, KPC-2, CTX-M-3, GES-1, IMP-25, OXA-1, OXA-50, OXA-10, PER-1, and TEM-1D) among the isolates (Fig. 2A and B). Except for the chromosome-encoded β-lactamases OXA-50 and AmpC, KPC-2 was the most prevalent β-lactamase. KPC-2 is one of the most effective carbapenemases and can inactivate all β-lactam antibiotics. Unfortunately, KPC-2 is only partially inhibited by β-lactamase inhibitors (40, 41). As demonstrated in this study, we observed 16-fold-higher MIC_50_s of carbapenems and higher rates of resistance (MIC_50_) to other β-lactams in *bla*_KPC-2_-positive CRPA isolates than in *bla*_KPC-2_-negative CRPA isolates (Table 1). KPC-type carbapenemases have been predominantly found in K. pneumoniae but are less frequent in P. aeruginosa. However, reports of KPC production among P. aeruginosa isolates have been increasing since the first detection of plasmid-borne KPC-2 in 2007 (42, 43). In P. aeruginosa, the metallo-β-lactamases (MBLs) are the most commonly detected carbapenemases, with VIM and IMP types being the most widely distributed geographically (19). In this study, however, KPC has become the predominant carbapenemase in P. aeruginosa. A novel combination of ceftazidime/avibactam was recently approved to treat infections by carbapenem-resistant Gram-negative bacteria (44). Encouragingly, it has improved the outcomes of infections caused by *Enterobacteriaceae*, which display resistance to carbapenems because of the production of class A β-lactamases, including KPCs; class C β-lactamases; and certain oxacillinases (i.e., OXA-48 carbapenemases) (45–49). In P. aeruginosa, it also demonstrated good *in vitro* activity against *bla*_KPC-2_-negative CRPA (50). It will be necessary to test ceftazidime/avibactam in *bla*_KPC-2_-positive CRPA isolates, especially clone ST463, in the future.

By genome sequencing, we found that a highly carbapenem-resistant clone (ST463) emerged and expanded in our hospital. Recently, it has spread to the neighboring hospitals in the city of Hangzhou, China (21). However, we found only a few ST463 isolates (*n* = 8) from other countries (including South Korea, France, Israel, and the United States) by searching the literature (51) and the NCBI genome database (see Table S4 in the supplemental material). None of them carried the resistance gene *bla*_KPC-2_. In contrast, the majority of ST463 isolates (92.2%) in this study contained the carbapenem resistance gene *bla*_KPC-2_ on plasmids pPA1011 and/or pRBL16. pPA1011 was the main vector of the KPC-2-encoding gene *bla*_KPC-2_ (Fig. 2C). The successful expansion of clone ST463 in China might be closely associated with *bla*_KPC-2_ in P. aeruginosa (Fig. 3C). Notably, the genetic context of *bla*_KPC-2_ on the plasmid was diverse by several insertion elements and transposons in proximity, but the IS*Kpn27*-*bla*_KPC-2_-IS*Kpn6* unit was conserved among all the isolates tested (Fig. 2D and Fig. S1). Besides pPA1011, we determined that the *bla*_KPC-2_ unit was carried by another plasmid, pRBL16, as well, and one isolate carried the *bla*_KPC-2_ unit in both plasmids pPA1011 and pRBL16. In addition, pPA1011 encoded a type IV secretion system (T4SS) for conjugation. All of these traits indicate that *bla*_KPC-2_ may be transferable with IS*Kpn27* and IS*Kpn6* between plasmids and strains, which might have favored the spread of KPC in P. aeruginosa. Genome-wide association analysis showed that 567 genes were highly associated with clone ST463, including several known virulence genes related to the biosynthesis of LOS O-antigens and exotoxin (35), although most of them (81%) remain functionally unknown (Fig. 4B and Table S3). In P. aeruginosa, the genomes are remarkably versatile, and clone-associated accessory genomes (including plasmids) commonly carry genes for antimicrobial resistance (AMR), a great diversity of metabolic pathways, and virulence factors (17). This allows P. aeruginosa to adapt to a wide range of niches. It needs to be further investigated how the associated genes contributed to the success of clone ST463 in the hospital environment. As we learned from the high-risk international clone ST235 of P. aeruginosa (18), a clone armed with a combination of virulence genes and high-level resistance is worrisome for public health. ST463 carries the plasmid-borne *bla*_KPC-2_ and virulence-related genes; thus, close surveillance should be undertaken in the future.

10.1128/mSystems.00154-21.7 TABLE S4 Genomes of Pseudomonas aeruginosa ST463 outside China. Download Table S4, XLSX file, 0.01 MB. Copyright © 2021 Hu et al. 2021 Hu et al. https://creativecommons.org/licenses/by/4.0/ This content is distributed under the terms of the Creative Commons Attribution 4.0 International license.

In summary, we analyzed the phenotypes and genotypes of CRPA isolates from 2006 to 2018 in one hospital located in Zhejiang, China, and found that an emergent clone, ST463, has become predominant in the CRPA population and is highly resistant to β-lactams, including carbapenems, and fluoroquinolones. Genome sequencing showed that plasmid-borne *bla*_KPC-2_ was associated with ST463 expansion, which conferred high-level resistance to carbapenems. The clinical use of β-lactam and/or carbapenem antibiotics to treat nosocomial infections may have driven this clonal expansion. The mobile elements flanking *bla*_KPC-2_, the type IV secretion system on the plasmid, and the successful expansion of clone ST463 may have favored *bla*_KPC-2_ spread in P. aeruginosa. Molecular clock analysis dated the emergence of clone ST463 to 2007. Genome-wide association analysis showed that 567 genes were highly associated with clone ST463, including several known virulence genes related to the biosynthesis of LOS O-antigens and exotoxin, but how the associated genes contributed to the success of clone ST463 in the hospital environment remains to be further investigated.

## MATERIALS AND METHODS

### Strains and antimicrobial susceptibility testing.

In this study, 544 carbapenem-resistant P. aeruginosa (CRPA) isolates were PCR screened for the most common carbapenemase genes as described previously, including *bla*_KPC_ (26), *bla*_NDM-1_ (52), *bla*_IMP_, and *bla*_VIM_ (53). The 544 nonduplicated CRPA isolates were collected from the Second Affiliated Hospital of Zhejiang University from 2006 to 2018 as a part of the Annual Review of Hospital Infection Resistance Survey in Zhejiang, China (22). Subsequently, a subset of isolates was randomly chosen from each year, totaling 143 isolates (105 *bla*_KPC_-positive isolates and 38 *bla*_KPC_-negative isolates), for antimicrobial susceptibility testing and whole-genome sequencing. Susceptibilities to 13 routinely used antimicrobial agents in clinical settings (imipenem, meropenem, ceftazidime, cefepime, piperacillin/tazobactam, cefoperazone/sulbactam, aztreonam, ciprofloxacin, levofloxacin, amikacin, gentamicin, colistin, and polymyxin B) were tested by the broth microdilution method according to the manufacturer’s instructions (Zhuhai DL, Shenzhen, China) and interpreted according to guideline document M100-S28 established by the Clinical and Laboratory Standards Institute (CLSI) (54). As no breakpoint was proposed for cefoperazone/sulbactam in P. aeruginosa by the CLSI, it was interpreted according to breakpoints for cefoperazone against *Enterobacteriales*.

### Genome sequencing.

Genomic DNA of all 143 isolates was extracted using a Wizard genomic DNA purification kit (Promega, Beijing, China), according to the manufacturer’s instructions. Indexed Illumina sequencing libraries were prepared using a TruSeq DNA PCR-free sample preparation kit (Illumina Inc., San Diego, CA) according to standard protocols. Libraries were sequenced on the Illumina HiSeq X10 platform by 150-bp paired-end strategies according to the manufacturer’s protocols (Bionova, Beijing, China). Raw reads were trimmed by Trimmomatic (55) and assembled into contigs using SPAdes v3.11.1 (56). Eight isolates (1120, 1704, 1708, 1709, 1619, 1814, 1217, and ZE5) were further sequenced using the Nanopore platform and assembled using Unicycler under the hybrid assembly mode (57).

### Molecular epidemiology, analysis of virulence and antibiotic resistance genes, genome-wide association study, and temporal analysis.

Assembled genomes were aligned, and a core-genome-based phylogenetic tree was generated by Parsnp in the Harvest package (58). MLST analysis and examination of known virulence-associated genes and antibiotic resistance genes were performed using the SRST2 pipeline, which takes Illumina reads as the input (59). Reference sequences of virulence genes and antibiotic resistance genes were obtained from the VFDB (60) and ARG-ANNOT (61) databases, respectively. The tree and the molecular features of each isolate were visualized using the online tool iTOL (62). Although we also found eight genomes of ST463 from other countries (see Table S4 in the supplemental material), we did not include them in the following genome-wide association study and temporal analysis because none of them carried the gene *bla*_KPC-2_, and the low numbers of genomes were insufficient to be representative geographically. To identify the genes associated with ST463, the 143 assembled genomes were first annotated using Prokka (63), the core genomes and accessory genomes were analyzed using Roary (64), and a pangenome-wide association analysis was then performed using Scoary (65). To date the last common ancestor of ST463, core genomes of the 77 ST463 isolates were aligned, and recombination regions were detected by the BratNextGen method (66). After the removal of recombinant segments, the temporal signal in the sequence data of ST463 was analyzed using TempEst (67). Whole-genome MLST (wgMLST) and core-genome MLST (cgMLST) of the 77 ST463 isolates were performed using chewBBACA (68) and visualized by using PHYLOViZ (69).

### Analysis of *bla*_KPC-2_ location and genetic context.

To detect the potential plasmids in the 143 isolates, Illumina reads were searched by PlasmidSeeker against the NCBI plasmid database (28). The reference sequences of the detected plasmids were then retrieved from the NCBI database (pPA1011, GenBank accession number MH734334; pRBL16, GenBank accession number CP015879.1). The presence of the reference plasmids was further confirmed by searching all the genes carried by each plasmid using SRST2 (59). *bla*_KPC-2_-containing contigs were extracted from the draft genomes and mapped to the reference pPA1011 or pRBL16 plasmid by BLASTN (70). The similarity of the flanking sequences of *bla*_KPC-2_ indicated that *bla*_KPC-2_ was carried by pPA1011 and/or pRBL16 in each isolate. To characterize the mobile genetic elements and antibiotic resistance genes in the immediate proximity of *bla*_KPC-2_, *bla*_KPC-2_-containing contigs were further annotated using RAST (71) and examined manually. The insertion sequences were identified using ISfinder (72).

### Ethics approval.

Ethical approval was obtained from the Ethics Committee of the Second Affiliated Hospital of Zhejiang University School of Medicine (approval number 2020-319). The strains used in this study were collected previously from routine microbiological specimens, while all the microbiological specimens were anonymized. Patients were not physically involved in this study. Therefore, no consent was needed for this study.

### Data availability.

The Illumina sequences generated and used in this study have been deposited and are available in the Sequence Read Archive (SRA) (http://www.ncbi.nlm.nih.gov/sra) under study accession number PRJNA648026. All 143 P. aeruginosa isolates are available under BioSample accession numbers SAMN15616606 to SAMN15616748. The genomes from Nanopore sequencing are available under GenBank accession numbers GCA_016807225.1, GCA_016820155.1, GCA_016820125.1, GCA_016820115.1, GCA_016820095.1, GCA_016820075.1, GCA_016820025.1, and GCA_016820015.1. All other data generated or analyzed during this study are included in this article and its additional files.
