# Supplementary figures and images for: Emergence and Expansion of a Carbapenem-Resistant Pseudomonas aeruginosa Clone Are Associated with Plasmid-Borne blaKPC-2 and Virulence-Related Genes
Source: mSystems. 2021 May 18;6(3):e00154-21. doi: 10.1128/mSystems.00154-21 (PMC8269210; doi:10.1128/mSystems.00154-21)

Figure S1

pPA1011

pRBL16

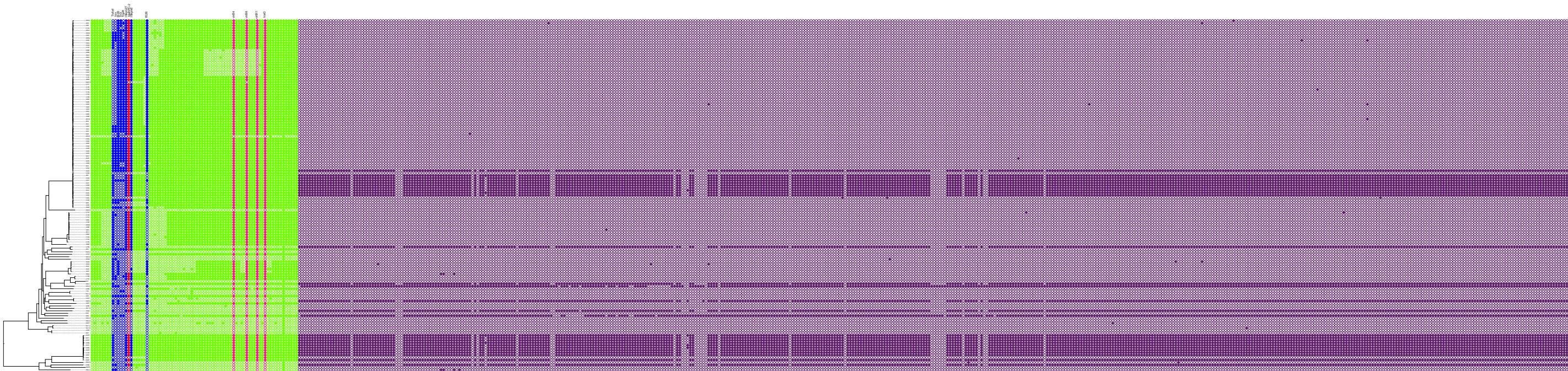

Supplement: FIG S1 [file msystems.00154-21-sf001.pdf]

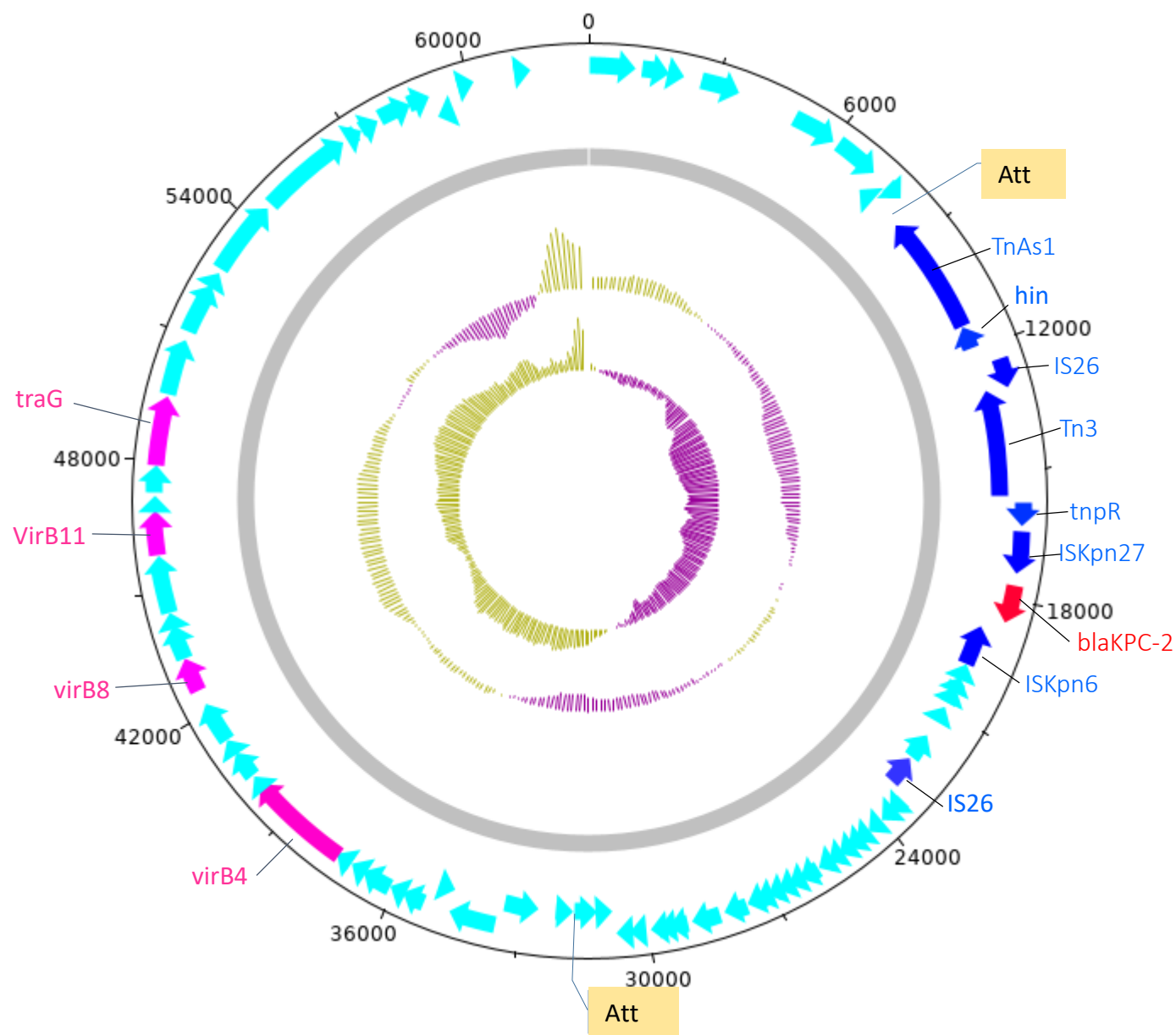

Figure S2

Supplement: FIG S2 [file msystems.00154-21-sf002.pdf]

A

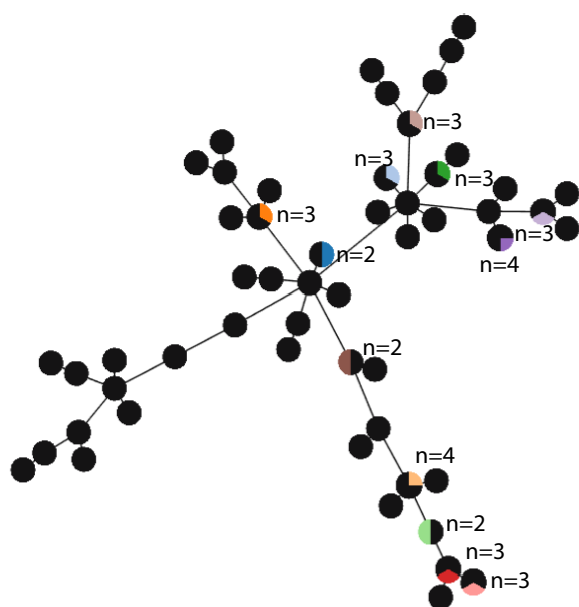

B

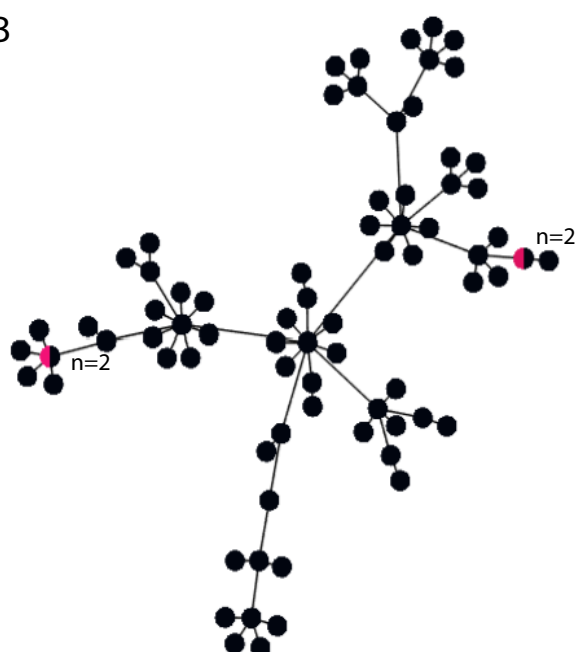

C

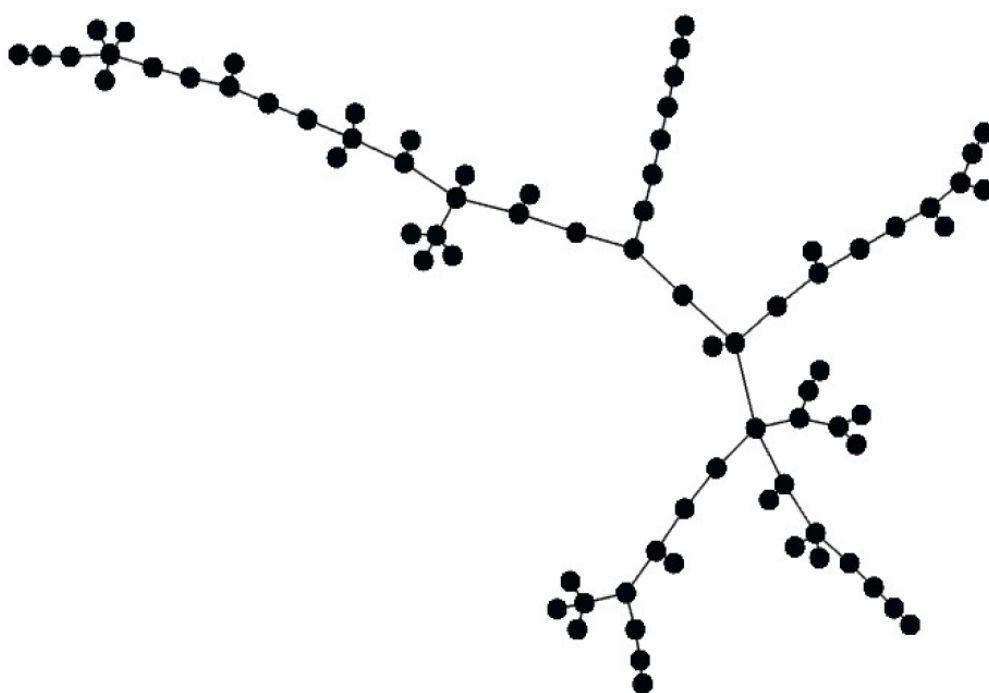

Figure S3

Supplement: FIG S3 [file msystems.00154-21-sf003.pdf]
